# Supplementary material for: Identification and reconstitution of the rubber biosynthetic machinery on rubber particles from Hevea brasiliensis
Source: eLife. 2016 Oct 28;5:e19022. doi: 10.7554/eLife.19022 (PMC5110245; doi:10.7554/eLife.19022)
Supplement: Figure 2—source data 1. — RP, rubber particle. DOI: http://dx.doi.org/10.7554/eLife.19022.004 [file elife-19022-fig2-data1.docx]

**Figure 2–source data 1. Proteins related to the natural rubber biosynthesis (upper list) and vesicular trafficking (lower list) identified in the RP proteomics.**

| **Family** | **Member** | **Accession** | **Score** | **Mass** | **Sequences** | **emPAI** | **Annotation** | **Predicted function** |
| --- | --- | --- | --- | --- | --- | --- | --- | --- |
| **Natural rubber biosynthesis-related proteins** | | | | |  |  |  |  |
| 1 | 1 | JT948852.1_c2 | 5669 | 21625 | 7 | 4.1 | REF/SRPP-family protein | RP stability |
| 2 | 1 | JT939480.1_c1 | 4652 | 26150 | 9 | 2.88 | REF/SRPP-family protein | RP stability |
| 2 | 2 | JT942555.1_c1 | 4567 | 23621 | 9 | 3.46 | REF/SRPP-family protein | RP stability |
| 3 | 1 | JT951018.1_2 | 2640 | 14713 | 8 | 5.55 | REF | NR biosynthesis |
| 5 | 1 | JT945746.1_2 | 2238 | 29535 | 15 | 11.59 | HRBP | NR biosynthesis |
| 8 | 1 | JT949604.1_3 | 1093 | 22331 | 9 | 3.15 | SRPP | RP stability |
| 12 | 1 | JT957715.1_2 | 482 | 17567 | 8 | 3.93 | REF/SRPP-family protein | RP stability |
| 15 | 1 | JT948343.1_1 | 399 | 19613 | 7 | 2.5 | REF/SRPP-family protein | NR biosynthesis |
| 20 | 1 | JT937325.1_3 | 296 | 33119 | 6 | 0.91 | HRT1/HRT2 | NR biosynthesis |
| 26 | 1 | JT946133.1_c3 | 213 | 24347 | 4 | 0.79 | REF/SRPP-family protein | RP stability |
| 27 | 1 | JT939205.1_c1 | 211 | 27055 | 6 | 1.2 | REF/SRPP-family protein | RP stability |
| 38 | 1 | JT942264.1_c1 | 133 | 25638 | 3 | 0.51 | REF/SRPP-family protein | RP stability |
|  |  |  |  |  |  |  |  |  |
| **Vesicular trafficking-related proteins** | | | |  |  |  |  |  |
| 4 | 1 | JT940643.1_c1 | 2301 | 41234 | 10 | 2.7 | endo-1,3-beta-glucosidase | Lipid body-associated enzyme |
| 10 | 1 | JT937869.1_c3 | 558 | 23973 | 7 | 1.81 | RABE1c | RabE/Rab8 (endosome) |
| 10 | 2 | JT944521.1_c3 | 519 | 23958 | 7 | 1.81 | RABE1c | RabE/Rab8 (endosome) |
| 10 | 3 | JT947009.1_3 | 517 | 24043 | 7 | 1.8 | RABE1c | RabE/Rab8 (endosome) |
| 10 | 4 | JT946657.1_3 | 508 | 24056 | 7 | 1.8 | RABE1c | RabE/Rab8 (endosome) |
| 10 | 5 | JT950598.1_c2 | 425 | 22771 | 5 | 1.54 | RABD1 | RabD/Rab1 (ER-Golgi) |
| 10 | 6 | JT945059.1_c1 | 357 | 22833 | 6 | 1.53 | RABD2c | RabD/Rab1 (ER-Golgi) |
| 10 | 7 | JT944033.1_c3 | 246 | 22796 | 4 | 0.86 | RABD2a | RabD/Rab1 (ER-Golgi) |
| 10 | 8 | JT941172.1_c1 | 241 | 23201 | 6 | 1.91 | RABH1b | RabH/Rab6 (Golgi) |
| 10 | 9 | JT945574.1_2 | 113 | 23912 | 2 | 0.35 | RABC1 | RabC/Rab18 (endosome) |
| 13 | 1 | JT945402.1_1 | 434 | 22048 | 11 | 6.98 | SAR1A | Coat-GTPase (ER-Golgi) |
| 13 | 2 | JT943935.1_c1 | 359 | 22140 | 8 | 3.94 | SAR1A | Coat-GTPase (ER-Golgi) |
| 18 | 1 | JT952583.1_c2 | 256 | 23563 | 6 | 1.46 | RABB1b | RabB/Rab2 (ER-Golgi) |
| 18 | 2 | JT941238.1_3 | 192 | 10671 | 4 | 2.58 | RABB1c | RabB/Rab2 (ER-Golgi) |
| 19 | 1 | JT943972.1_1 | 300 | 23324 | 9 | 3.56 | RABG3f | RabG/Rab7 (endosome, vacuoles) |
| 19 | 2 | JT944915.1_c2 | 260 | 23297 | 8 | 2.92 | RABG3f | RabG/Rab7 (endosome, vacuoles) |
| 29 | 1 | JT944240.1_c3 | 198 | 24305 | 5 | 1.07 | RABA1c | RabA/Rab11 (endosome) |
| 29 | 2 | JT948813.1_3 | 189 | 22056 | 5 | 1.22 | RABF1 | RabF/Rab5 (endosome) |
| 29 | 3 | JT945020.1_1 | 177 | 18166 | 4 | 1.16 | RABF1 | RabF/Rab5 (endosome) |
| 29 | 4 | JT945065.1_1 | 140 | 21871 | 3 | 0.62 | RABF2a | RabF/Rab5 (endosome) |
| 29 | 5 | JT950302.1_1 | 119 | 24028 | 4 | 0.8 | RABA2a | RabA/Rab11 (endosome) |
| 39 | 1 | JT933735.1_c1 | 128 | 25104 | 2 | 0.33 | RABA4c | RabA/Rab11 (endosome) |
| 53 | 1 | JT938614.1_2 | 95 | 23702 | 2 | 0.35 | RABC1 | RabC/Rab18 (endosome) |
| 54 | 1 | JT946100.1_c2 | 93 | 21378 | 1 | 0.18 | Clathrin light chain | Coat complex Clathrin (TGN, endosome) |
| 72 | 1 | JT948536.1_c2 | 55 | 22740 | 1 | 0.17 | VAMP-like protein YKT61 | R-SNARE/VAMP (TGN) |
| 75 | 1 | JT914456.1_c2 | 52 | 194165 | 1 | 0.02 | Clathrin heavy chain 1 | Coat complex Clathrin(TGN, endosome) |
| 76 | 1 | JT941244.1_c3 | 51 | 21863 | 1 | 0.18 | ARFB2a | Coat-GTPase (endosome, ER-Golgi) |
| 86 | 1 | JT944943.1_3 | 43 | 24986 | 2 | 0.33 | RABA4a | RabA/Rab11 (endosome) |
