# Supplementary material for: Identification and reconstitution of the rubber biosynthetic machinery on rubber particles from Hevea brasiliensis
Source: eLife. 2016 Oct 28;5:e19022. doi: 10.7554/eLife.19022 (PMC5110245; doi:10.7554/eLife.19022)
Supplement: Supplementary file 1. — DOI: http://dx.doi.org/10.7554/eLife.19022.026 [file elife-19022-supp1.docx]

**Supplementary file 1. List of primers used in this study.**

| **Experiment** | **Primer name** | **Sequence** |
| --- | --- | --- |
| Cloning of a cDNA for *HRBP* | HbNgBR-Fw | 5'-ATGGATTTGAAACCTGGAGCTGG-3', |
|  | HbNgBR-Rv | 5'-TTATGTACCATAATTTTGCTGCACTGTTG-3' |
| Quantitative Real-time Reverse transcription PCR | HRBP-Fw | 5′-AATTTGCTTCCAGCTCCGATGG-3′ |
|  | HRBP-Rv | 5′-TAGATGGCATCTAACAGGTCC-3′ |
|  | 18SrRNA-S | 5′-CAAAGCAAGCCTACGCTCTG-3′ |
|  | 18SrRNA-A | 5′-TGCTTTCGCAGTTGTTCGTC-3′ |
| Split-ubiquitin-based Y2H assay and library screening | REF-Fw | 5'-ATTAACAAGGCCATTACGGCCATGGCTGAAGACGAAGACAACCAAC-3' |
|  | REF-Rv | 5'-AACTGATTGGCCGAGGCGGCCCCA TTCTCTCCATAAAACACCTTAGCAAG-3' |
|  | pPR3N Sequence Forward Primer | 5'-GTCGAAAATTCAAGACAAGG-3' |
|  | pPR3N Sequence Reverse Primer | 5'-AAGCGTGACATAACTAATTAC-3' |
|  | HRT1/HRT2 Rv2 Y2H | 5'-TTTGGCCGAGGCGGCCTTATTTTAAGTATTCCTTATGTTTC-3' |
|  | NgBR Fw Y2H | 5'-TTTGGCCATTACGGCCATGGATTTGAAACCTGGAG-3' |
|  | NgBR Rv2 Y2H | 5'-TTTGGCCGAGGCGGCCTCATGTACCATAATTTTGCTGCAC-3' |
|  | N-REF-SfiI_Fw | 5'-ATGGGCCATTACGGCCATGGCTGAAGACGAAGACAAC-3' |
|  | N-REF-SfiI_Rv | 5'-TAGGCCGAGGCGGCCTCAATTCTCTCCATAAAACACCTTA-3' |
|  | N-SRPP-SfiI_Fw | 5'-ATGGGCCATTACGGCCATGGCTGAAGAGGTGGAG-3' |
|  | N-SRPP-SfiI_Rv | 5'-ATAGGCCGAGGCGGCCTTATGATGCCTCATCTCCAAAC-3' |
| BiFC assay and subcellular localization analyses | MCS1-REF-NcoI_Fw | 5'-ATGCCATGGCTGAAGACGAAGACAAC-3' |
|  | MCS1-REF-SpeI_Rv | 5'-ATGACTAGTATTCTCTCCATAAAACACCTTAGC-3' |
|  | MCS1-HRT1-NcoI_Fw | 5'-ATGCCATGGAATTATACAACGGTGAGAG-3' |
|  | MCS1-HRT1-SpeI_Rv | 5'-ATGACTAGTTTTTAAGTATTCCTTATGTTTCTCCAAG-3' |
|  | MCS1-HRBP-NcoI_Fw | 5'-ATGCCATGGATTTGAAACCTGGAGC-3' |
|  | MCS1-HRBP-SpeI_Rv | 5'-ATGACTAGTTGTACCATAATTTTGCTGCAC-3' |
|  | MCS3-REF-RsrII_Fw | 5'-ATGCGGTCCGCTGAAGACGAAGACAACC-3' |
|  | MCS3-REF-AatII_Rv | 5'-ATGGACGTCACATTCTCTCCATAAAACACCTTAGC-3' |
|  | MCS3-HRBP-RsrII_Fw | 5'-ATGCGGTCCGATTTGAAACCTGGAGCTGG-3' |
|  | MCS3-HRBP-AatII_Rv | 5'-ATGGACGTCACTGTACCATAATTTTGCTGCAC-3' |
|  | mCherry-BamHI_Fw | 5'-ATAGGATCCGTGAGCAAGGGCGAGG-3' |
|  | mCherry-XbaI_Rv | 5'-AGCTCTAGATTACTTGTACAGCTCGTCCATG-3' |
|  | MCS3-HRBP-AatII_Rv | 5'-ATGGACGTCTTATGTACCATAATTTTGCTGCAC-3' |
| Protein expression with the wheat germ cell-free system | CF-HRT Fw | 5'-TTTGGATCCGATGGAATTATACAACGGTGAGAGG-3' |
|  | CF-HRT Rv | 5'-TTTGCGGCCGCTTATTTTAAGTATTCCTTATGTTTCTCC-3' |
|  | CF-REF Fw | 5'-TTTCTCGAGATGGCTGAAGACGAAGAC-3' |
|  | CF-REF Rv Stop | 5'-TTTGGATCCTCAATTCTCTCCATAAAAC-3' |
|  | CF-HRBP Fw | 5'-TTTCTCGAGATGGATTTGAAACCTGGAGCTG-3' |
|  | CF-HRBP Rv | 5'-TTTCTCGAGTGTACCATAATTTTGCTGCAC-3' |
